# Quality Improvement Initiative to Increase Rate of and Time to Post-intubation Analgesia in the Emergency Department

**DOI:** 10.5811/westjem.2021.4.51115

**Published:** 2021-07-14

**Authors:** Bryan Imhoff, Samuel J. Wagner, Kelly Howe, Jonathan Dangers, Niaman Nazir

**Affiliations:** * The University of Kansas Health System, Department of Emergency Medicine, Kansas City, Kansas; † The University of California San Francisco, Department of Emergency Medicine, San Francisco, California

## Abstract

**Introduction:**

Intubation and mechanical ventilation are common interventions performed in the emergency department (ED). These interventions cause pain and discomfort to patients and necessitate analgesia and sedation. Recent trends in the ED and intensive care unit focus on an analgesia-first model to improve patient outcomes. Initial data from our institution demonstrated an over-emphasis on sedation and an opportunity to improve analgesic administration. As a result of these findings, the ED undertook a quality improvement (QI) project aimed at improving analgesia administration and time to analgesia post-intubation.

**Methods:**

We performed a pre-post study between January 2017–February 2019 in the ED. Patients over the age of 18 who were intubated using rapid sequence intubation (RSI) were included in the study. The primary outcome was the rate of analgesia administration; a secondary outcome was time to analgesia administration. Quality improvement interventions occurred in two phases: an initial intervention focused on nursing education only, and a subsequent intervention that included nursing and physician education.

**Results:**

During the study period, 460 patients were intubated in the ED and met inclusion/exclusion criteria. Prior to the first intervention, the average rate of analgesia administration was 57.3%; after the second intervention, the rate was 94.9% (P <0.01). Prior to the first intervention, average time to analgesia administration was 36.0 minutes; after the second intervention, the time was 16.6 minutes (P value <0.01).

**Conclusion:**

This QI intervention demonstrates the ability of education interventions alone to increase the rate of analgesia administration and reduce the time to analgesia in post-intubation patients.

## INTRODUCTION

Rapid sequence intubation (RSI) and mechanical ventilation are common interventions performed in the emergency department (ED). These interventions cause pain and discomfort to patients.^1^,^2^ Patients generally require pharmacologic interventions to tolerate ongoing mechanical ventilation. These medications are generally categorized as analgesics or sedatives.

Multiple studies have demonstrated risks with excessive sedation. A landmark study in 2000 by Kress et al coined the term “sedation vacation” and correlated reduced sedation with decreased days spent on the ventilator and in the intensive care unit (ICU).^3^ Further studies demonstrated a relationship between deep sedation and worse patient outcomes including delayed extubation, increased delirium, and increased mortality.^4^,^5^ A follow-up, multicenter, randomized controlled trial indicated that goal-directed sedation was “feasible, appeared safe, achieved early light sedation, minimized benzodiazepines and propofol, and decreased the need for physical restraints.”^6^ The risks of sedation extend to the ED, with one prospective cohort study showing a significant mortality association with “early deep sedation” in patients intubated in the ED.^7^

Recent critical care literature has shown that minimizing sedation via development of a nursing or pharmacist protocol leads to improvement in patient-centered outcomes such as decreased number of intubated days and decreased hospital length of stay.^8^,^9^ Research also suggests that sedation can be minimized by switching to an analgesia-first model. A comparative study in Cambridge, UK, showed that protocols emphasizing analgesia can lower sedation requirements for mechanically ventilated patients.^10^ Additional studies demonstrate similar findings, including one ICU clinical trial.^11^,^12^

An initial analysis of the use of post-intubation pharmacologic agents in our institution’s ED indicated an overemphasis on sedation and an opportunity to increase analgesic administration. We collected data on a sample of 390 intubated and mechanically ventilated patients between January 2016–October 2017 in the ED. During this period, 30% of patients received sedation without analgesia and 13% received neither analgesia nor sedation, seemingly inconsistent with the research presented above that demonstrates improved patient outcomes with analgesia followed by light, goal-directed sedation. As a result of these initial findings, the ED undertook a quality improvement (QI) project aimed at improvement of analgesia administration and time to analgesia post-intubation.

## METHODS

### Study Design

This pre-post interventional study evaluated the rate of analgesia administration and time to analgesia following RSI in the ED. This study evaluated outcomes both prior to and following two separate interventions. As a QI project, this study was deemed exempt from institutional review board approval.

### Study Setting

This study was conducted at a large, academic, tertiary care center in the Midwest.

### Patient Selection

Patients over the age of 18 who were intubated in the ED using RSI from January 2017 –February 2019 were included in the study. Both induction and paralytic agents must have been given to the patients to make them eligible for participation.. We excluded patients who were in cardiac arrest or profound shock (defined as mean arterial pressure < 65 millimeters mercury in the peri-intubation phase of care and/or those on vasopressors). We also excluded patients who were trauma activations because initial resuscitation for these patients is managed jointly between the ED and trauma team at this institution, and our intervention efforts were designed to target ED staff only.

**Population Health Research Capsule**

What do we already know about this issue?

*Excessive sedation has been correlated with negative patient outcomes post-intubation; emphasizing analgesia has been shown to reduce patient sedation requirements*.

What was the research question?

*Can a quality improvement project increase the rate of analgesia administration and reduce time to analgesia post-intubation?*

What was the major finding of the study?

*A cross-functional education intervention successfully improved both measures*.

How does this improve population health?

*Increasing timely analgesia post-intubation can help reduce sedation requirements, a recognized contributor to negative patient outcomes*.

### Data Collection and Measures

A dataset of patients meeting inclusion criteria was generated via a query of our electronic health record (EHR) system Epic (Epic Systems Corporation, Verona, WI). We collected data on the date, time, and medications given in the peri-intubation phase of care. For each patient, the induction and paralytic agents were identified, and we recorded the first analgesic and/or sedative given after intubation. Induction agents included etomidate, ketamine, propofol, and midazolam. Paralytic agents included rocuronium, succinylcholine, and vecuronium. The first dose of fentanyl or ketamine after the induction agent, if given, was recorded as an analgesic agent. The first dose of propofol, midazolam, ketamine, lorazepam, or dexmedetomidine, if given, was recorded as a sedative agent.

After collecting the data, we calculated how many patients received no analgesia; analgesia only; no sedation; sedation only; and those who received both analgesia and sedation. We also calculated the time to administer the analgesic from the time the induction agent was given. As a subanalysis, we were interested in the subset of patients who received rocuronium during RSI, as these patients experience longer durations of paralysis, which can have implications on timing of analgesia and sedation.

### Interventions

During the study period, two interventions were completed: a nursing-only education intervention in November 2017 followed by a broader physician and nursing education intervention in May 2018. A systems improvement, in the form of a new EHR order-set that centralized post-intubation analgesia and sedation options with laboratory and imaging orders (eg, post-intubation chest radiograph [CXR] and arterial blood gas) was initially planned as part of the second intervention; however, due to information technology (IT) delays, this systems improvement was developed and implemented later. The order-set was ultimately implemented in February 2019 after our post-implementation evaluation period. Any improvement tied to the systems enhancement was intentionally not included in our results below. For the purposes of this study, the pre-intervention timeframe included patients from January 1–October 31, 2017, post-intervention 1 from December 1, 2017–April 30, 2018, and post-intervention 2 from June 1, 2018– February 28, 2019.

The first intervention included nursing-only education focused broadly on all elements of intubation and occurred between November 1–November 30, 2017. During this period, all ED nurses were required to complete education and could choose from an in-person class or self-study with a subsequent test. Thirty-one nurses chose to attend in-person, and 94 chose self-study. Topics in person and via self-study were identical and are included in Table 1. A test of proficiency was created in house; nurses were required to score 80% or better and could retake the test until achieving that score.

The second intervention targeted education of both nurses and physicians and took place during May 2018. For the nursing staff, the second intervention served as an opportunity to review the material described above. Seventy ED nurses attended an in-person class; understanding was again tested using the identical online test of proficiency. Physician education interventions focused more heavily on residents (than attendings) and included the following: (1) one hour off-line, self-study topic for residents using outside sources in preparation for weekly didactics on May 23, 2018; (2) journal club discussion on the topic during weekly didactics on May 23, 2018; and (3) an interactive live presentation reviewing ED post-intubation analgesia and sedation performance during resident didactics on May 23, 2018. Attending physicians received the same presentation (#3 described above) during the May 2018 ED faculty staff meeting. Finally, the findings from #3 were summarized and emailed to all resident and attending physicians for offline review. Physician interventions did not include a test of understanding/proficiency. Table 2 provides a detailed list of source materials for resident didactics and journal club.

These interventions were undertaken with the support but not the mandate of departmental and residency leadership. We did not analyze the performance or behavioral change of individual providers as part of this project. Moreover, providers were informed that aggregate, rather than individual, performance would be reported. There were no additional incentives, explicit or implicit, for providers to implement these changes.

### Statistics

We summarized categorical variables with frequency and percentages. Due to non-normal distribution, continuous variables were summarized by means, medians and interquartile range. We tested associations between categorical variables using chi-square test. We used analysis of variance and, where appropriate, we used non-parametric Wilcoxon rank-sum test and Kruskal-Wallis test to make global comparisons of continuous variables across groups. Two-sided *P*-values less than 0.05 were considered statistically significant. Data management and statistical analyses were performed using SAS software (version 9.4) (SAS Institute Inc., Cary, NC).

## RESULTS

A total of 192 intubations occurred during the pre-intervention period, 90 during post-intervention period 1, and 178 during post-intervention period 2. Patient characteristics are shown in Table 3. Vital signs represent first recorded after intubation. Analysis showed a statistical difference in paralytic used across the three study time periods. Analysis otherwise showed no statistically significant difference between patients in each group for the characteristics collected.

Table 4 presents rates of analgesia and/or sedation. Data includes the number of patients in each group prior to any intervention and after each intervention. The rate for groups 2 (analgesia without sedation) and 4 (analgesia and sedation) increased after each intervention, whereas a reverse trend was seen in the other groups (*P*-value <0.01).

**Figure 1:**
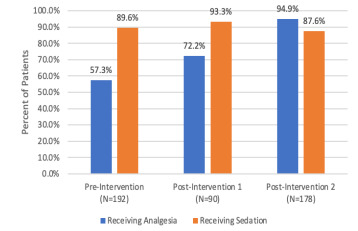
Percent of patients receiving analgesia, sedation.

Given a focus on analgesia administration in this QI project, Figure 1 summarizes total analgesia rate for each time period. Total sedation rate is included for comparison. The percent of intubated patients receiving analgesia increased after each intervention. This improvement in analgesia administration rate was statistically significant (*P* <0.01). Sedation rate minimally increased after the first intervention and then decreased after the second intervention. However, these changes in sedation rates were not statistically significant (*P =* 0.35).

Statistically, there was no difference in rates of analgesia (*P =* 1.0) between months 1–2 (95%) and months 8–9 (95%) during the post-intervention 2 time period. Similarly, there was no difference in rates of sedation (*P =* 0.14) between the same months (95% and 85%, respectively).

**Figure 2:**
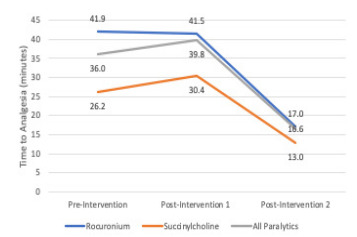
Time to analgesia (induction agent to analgesic agent).

In addition to improving analgesia rate, this QI project aimed to improve the time to analgesia administration (time from administration of induction agent to administration of analgesic agent). Comparisons were made pairwise between pre-intervention and post-intervention groups. Figure 2 summarizes these comparisons. For all paralytics, time to analgesia increased comparing pre-intervention and post-intervention 1 groups (36.0 minutes to 39.8 minutes, respectively) but was not statistically significant (*P =* 0.27). For all paralytics, time to analgesia decreased comparing post-intervention 1 and post-intervention 2 groups (39.8 minutes to 16.6 minutes, respectively) and was statistically significant (*P* <0.01). Finally, for all paralytics, time to analgesia also decreased comparing pre-intervention and post-intervention 2 groups (36 minutes to 16.6 minutes, respectively) and was also statistically significant (*P* <0.01).

Figure 2 also breaks down time to analgesia by agent for each time period. After both interventions, time to analgesia for rocuronium-induced intubations decreased (41.9 minutes vs 17.0 minutes) and succinylcholine-induced intubations decreased (26.2 minutes vs 13.0 minutes).

## DISCUSSION

Following the first intervention, the rate of analgesia administration increased from 57.3% to 72.2% as a result of nursing-focused education.^15^ Despite demonstrating an improvement, the magnitude of change was smaller than desired. Moreover, time to analgesia demonstrated no statistically significant change. In examining the intervention, impediments to improvement were thought to be as follows:

1. *Narrow scope*. Training during the first intervention was limited to nursing staff and excluded other material stakeholders, namely resident and attending physicians.
2. *Ordering complexity*. The ordering process (via EHR) required ordering medications for RSI and post-sedation care individually or using multiple order-sets.
3. *Inconsistent pain assessment*. The existing ventilator pain assessment tool seemed to be inconsistently used and rarely documented by nursing.

Interestingly, providers’ average choice of paralytic agent before and after the first intervention were statistically different. This nursing-focused intervention did not favor or emphasize one paralytic over another. We assume that rather than being the result of the intervention, this change in behavior correlates with the availability of rocuronium’s reversal agent sugammadex in our ED. However, the increasing use of rocuronium does create additional complexity in post-intubation analgesia and sedation. Theoretically, delays in pharmacologic administration could occur as paralysis is mistaken for lack of agitation or pain. One medical center noted a delay in administration of analgesia or sedation by about 30 minutes on average post-intubation following use of rocuronium.^16^ The duration of action of rocuronium can likely explain this discrepancy, as typical triggers for sedation and analgesia are blocked by the longer acting paralytic. One ED in Tucson, AZ, was able to use a pharmacist-led education program to eliminate this delay.^17^

Based on the small magnitude of change following our first intervention, a second intervention was intended to address the shortcomings identified above, first by broadening the scope of education and training to include physicians. Second, the intervention intended to simplify the ordering process through the creation of a new single EHR order-set that centralized post-intubation analgesia and sedation options and included RSI medication orders with related laboratory and imaging orders (eg, post-intubation CXR and arterial blood gas). As discussed above, due to IT delays, this improvement was rolled out later as a third intervention. The order-set was ultimately implemented in February 2019 after our post-implementation evaluation period. Any improvement tied to this system enhancement is not included in our results. Moreover, outside of the study authors and the department chair, no participating nurse, resident, or attending physician was involved in or aware of the planned order-set, thus limiting any confounding effect. Finally, the second intervention intended to reinforce (during a second round of nursing training) the hospital process and tool for ventilator pain assessment. These included not only more consistently assessing patient pain but also an emphasis on better documentation.

Following the second intervention, the rate of analgesia administration increased to 94.9%, and time to analgesia improved from 36.0 minutes pre-intervention to 16.6 post-intervention. This represents an improvement in both primary and secondary variables. Further, analysis comparing the first two to the last two months of this period demonstrated no statistically significant fatigue in adherence to training.

Some of the key factors that contributed to the ultimate success of this project included establishing a multidisciplinary team that included representatives from each major stakeholder group including nursing, pharmacists, resident physicians, and attending physicians. While the first intervention was narrowly focused on nursing education, a second broader intervention built on and expanded this initial work; a third intervention will incorporate EHR/systems changes. Key to the success of this project was using an iterative cycle to conduct multiple tests of change. This approach is known as the plan-do-study-act (PDSA) cycle. “[PDSA] cycles are the building blocks of iterative healthcare improvement. Each cycle combines prediction with a test of change (in effect, hypothesis testing), analysis and a conclusion regarding the best step forward—usually a prediction of what to do for the next PDSA cycle.”^18^ Finally, a balanced set of interventions targeting people, process, and technology was central in driving success.

## LIMITATIONS

A primary limitation of this QI effort was that the study’s data collection periods were unequal, subjecting results to potential differing effects of seasonality and potential differing degrees of adherence to training. Second, the study’s key outcome variables did not directly measure clinical outcomes. Measuring a primary patient outcome such as time to target pain score (eg, Critical Care Pain Observation Tool score) would be preferable but was problematic due to incomplete and/or inaccurate data). A subsequent QI project could target improving the capture and reporting of this data. Additionally, measuring primary patient outcomes such as post-extubation assessment of pain during a period of intubation and mechanical ventilation was designed to be out of scope due to the logistical difficulty and cost to collect such data. That said, based on the research cited in the introduction to this manuscript, we believe faster and more complete analgesia leads to improved patient experience and outcomes.

Third, the study demonstrated no statistical change in the rates of sedation before and after intervention but did not report the effect of the QI intervention on time to sedation. Theoretically, a focus on time to analgesia could have an unintended consequence on time to sedation. Third, the study excluded trauma activations from the study during the design phase due to dual management of these patients between ED and trauma teams. In addition, because this was a retrospective chart review the results are subject to potential issues related to validity and reliability inherent to this study type, including inaccurate or incomplete information in the medical chart. As a non-blinded, pre-post study, the results are subject to the Hawthorne effect and lack of comparison arm inherent to this study type. Finally, this study was performed at a single hospital and single ED, which inherently limits its generalizability.

## CONCLUSION

This quality improvement initiative was successful in increasing the rate of analgesia administration and reducing the time to analgesia in post-intubation patients in a single academic ED. The use of an iterative, plan-do-study-act process yielded improvements after each intervention. Areas for further study would include (1) assessing the impact of a new EHR order-set on the study’s primary variables and (2) determining the clinical significance of improving rates of analgesia and time to analgesia.

## Figures and Tables

**Table 1:** Nursing training topics.

Rapid sequence intubation (RSI) 7 Ps of RSI (preparation, preoxygenation, pretreatment, paralysis, protection, placement, post-intubation management) RSI medications (induction agents, paralytics) The failed airway Detailed post-intubation management Analgesia and sedation Medications (analgesics, sedatives) Ventilator management

**Table 2:** Physician training topics.

Off-line, Self-Study Topics	A New Paradigm for Post-Intubation Pain, Agitation and Delirium (PAD)^13^ Management of Pain, Agitation and Delirium in the ICU^14^
Journal Club Articles	Analgosedation Practices and the Impact of Sedation Depth on Clinical Outcomes Among Patients Requiring Mechanical Ventilation in the ED: A Cohort Study.^7^ Impact of an Analgesia-Based Sedation Protocol on Mechanically Ventilated Patients in a Medical Intensive Care Unit^11^

*ICU*, intensive care unit; *ED*, emergency department.

**Table 3:** Patient characteristics.

Characteristic	Pre-intervention (N = 192)	Post-intervention 1 (N = 90)	Post-intervention 2 (N = 178)	P-value **
Age (years)	58.5, 60 (20.5)	60.6, 61.5 (26)	59.4, 60.5 (21)	0.60
Male (%)	55.7	52.2	51.1	0.66
Weight (kg)	84.7, 80.7 (32.1)	81.2, 76 (31.8)	80.5, 78.7 (32)	0.23
Induction Agent (%)				0.33
Etomidate	84	82	83	
Ketamine	13	11	12	
Propofol	2	7	4	
Midazolam	2	0	1	
Paralytic Agent (%)				<0.01
Rocuronium	68	88	91	
Succinylcholine	32	12	9	
Post intubation Systolic (mm Hg)	141, 138 (45)	147, 148 (51)	148, 142 (49)	0.29
Post-intubation Diastolic (mm Hg)	85, 85 (33)	88, 88 (32)	91, 88 (31)	0.06
Post-intubation Mean Arterial Pressure (mm Hg)	98, 98 (35)	102, 101 (26)	106, 102 (32)	0.09
Post-intubation Heart Rate (per minute)	107, 107 (39)	108, 109 (27)	110, 107 (39)	0.61
Post-intubation Respiratory Rate (per minute)	18, 17 (6)	19, 18 (5)	18, 17 (6)	0.48
Post-intubation SpO_2_ (percent)	97.3, 99 (2)	98.4, 100 (1)	98.2, 100 (1)	0.21

-- Mean, median (interquartile range) unless specified otherwise.

** P-values based on analysis of variance. Gender and paralytic agent P-values are based on chi-square test. Induction agent P-value is based on Fisher’s exact test.

*kg*, kilograms; *mm Hg*, millimeters mercury; *SpO_2_*, oxygen saturation.

**Table 4:** Rates of analgesia and sedation.

Administration of Analgesia and/or Sedation	Pre-intervention (N = 192)	Post-intervention 1 (N = 90)	Post-intervention 2 (N = 178)
No Analgesia or Sedation	16 (8.33%)	3 (3.33%)	2 (1.12%)
Analgesia without Sedation	4 (2.08%)	3 (3.33%)	20 (11.24%)
Sedation without Analgesia	66 (34.38%)	22 (24.44%)	7 (3.93%)
Analgesia and Sedation	106 (55.21%)	62 (68.89%)	149 (83.71%)
